# Relationship between the psychosocial impact of dental aesthetics and perfectionism and self-esteem

**DOI:** 10.4317/jced.54481

**Published:** 2017-12-01

**Authors:** Alina Venete, Eva Trillo-Lumbreras, Vicente-Javier Prado-Gascó, Carlos Bellot-Arcís, José-Manuel Almerich-Silla, José-María Montiel-Company

**Affiliations:** 1 Grado en odontología [equivalent to BSc Dentistry], University of Valencia; 2 Post-Doctoral Teaching Assistant, Department of Social Psychology, University of Valencia; 3 Post-Doctoral Teaching Assistant, Department of Stomatology, Faculty of Medicine and Dentistry, University of Valencia; 4 Tenured Lecturer, Department of Stomatology, Faculty of Medicine and Dentistry, University of Valencia; 5 Teaching Assistant, Department of Stomatology, Faculty of Medicine and Dentistry, University of Valencia

## Abstract

**Background:**

Awareness of the influence of personality traits such as self-esteem and perfectionism on the aesthetic self-image can help clinicians to improve their patients’ satisfaction and quality of life. The main objective of this study was to examine the relationship between self-esteem, perfectionism and the psychosocial impact of dental aesthetics, and their association with gender.

**Material and Methods:**

A descriptive-analytical cross-sectional study was conducted in a sample of 301 students of the Faculty of Medicine and Dentistry of the University of Valencia, aged between 18 and 30 years. Each participant was asked to complete a survey comprising three questionnaires: PIDAQ (Psychosocial Impact of Dental Aesthetics Questionnaire), MPS (Multidimensional Perfectionism Scale) and RSS (Rosenberg Self-esteem Scale). The response rate was 79%.

**Results:**

The mean age was 20.8 years; 226 were women (75 %) and 75 were men (25 %). A negative correlation (Pearson = -0.387) was found between the total PIDAQ score and self-esteem. The correlation with perfectionism was found to be positive (Pearson = 0.281). On comparing the questionnaire and subscale scores by gender, the only statistically significant differences were in perfectionism (men 97.4, women 89.1) and self-confidence (men 22.1, women 23.5).

**Conclusions:**

The students most affected by poor dental aesthetics had lower self-esteem and higher levels of perfectionism. The men presented higher levels of perfectionism than the women, while the latter displayed greater self-confidence in their dental aesthetics. Clinicians should pay greater attention to these traits and to their implications for treating these patients.

** Key words:**Psychosocial impact of dental aesthetics questionnaire, multidimensional perfectionism scale, rosenberg self-esteem scale, students.

## Introduction

People’s self-image and perception of their dental aesthetics affect their social and psychological welfare, and this is reflected in their behaviour and self-confidence (1).

In the field of dentistry, greater attention has traditionally been paid to assessing malocclusion and dental aesthetics from an objective viewpoint, ignoring aspects that may impinge on the patient’s quality of life. The instruments most often used to assess malocclusion and dental aesthetics are the Dental Aesthetic Index (DAI) and the Index of Orthodontic Treatment Need – Aesthetic Component (IOTN-AC) (2,3). In recent years, as greater interest has been paid to the need for an instrument to measure aspects of dental aesthetics related to quality of life, the PIDAQ (Psychosocial Impact of Dental Aesthetics Questionnaire) has made its appearance (4).

Traditionally, one of the most important aspects of welfare or quality of life has been self-esteem (5). Self-esteem expresses the feeling that one is ‘good enough’. Individuals with self-esteem simply feel that they are of worth and respect themselves for what they are, but do not stand in awe of themselves or expect others to stand in awe of them.

It would appear to have been proved that self-esteem influences all aspects of a person’s life, from interpersonal relationships and satisfaction at work to aspects related to health and depression (6), and is one of the most important psychological factors that drive people to take decisions, relate to each other, reach a certain level of academic achievement or experience and express particular emotional reactions (7,8). In the field of health, self-esteem has also been related to treatment of a disease, compliance with the treatment and the prognosis of the disease (9).

One aspect that appears to have a substantial influence on the self-esteem of individuals, and therefore on their welfare and quality of life, is their self-image. As part of this self-image, particular attention has been paid in recent years to what is known as dental aesthetics, particularly when the person is dissatisfied with it (10). The bibliography suggests that those who are satisfied with the appearance of their face have higher self-esteem (10,11).

In the same way, poor dentofacial aesthetics has been associated with a greater psychosocial impact (12) and lower self-esteem in those who suffer from it (13,14). Self-esteem, as explained above, consists in how the person assesses his or herself. The tool most often used to measure it is the Rosenberg Self-esteem Scale (RSS). This questionnaire, which measures self-esteem in terms of feelings of personal worth and respect for oneself, has been widely used (15). Romero-Maroto *et al.* (16) found a negative correlation between the psychosocial impact of aesthetics and self-esteem.

Together with self-esteem, other psychological aspects appear to be related to dental aesthetics and could have a negative effect on both quality of life in general and levels of self-esteem in particular. One such aspect is perfectionism. Perfectionism is part of an individual’s personality and is another important trait to bear in mind, owing to its possible consequences. When greater than normal it can affect a person’s mental health, causing imbalance and constant distress (17). The MPS (Multidimensional Perfectionism Scale) developed by Frost *et al.* (18) can be used to assess perfectionism.

As well as these psychological aspects, other psychosocial factors, particularly age, gender and educational level, appear to influence both people’s satisfaction with their appearance and their self-esteem and levels of perfectionism (19,20).

Despite the importance of the psychological aspects, few authors have studied their association with the psychosocial impact of dental aesthetics, and most have focused on self-esteem but have not simultaneously considered perfectionism. A knowledge of the psychological aspects that influence the aesthetic self-image of patients can help clinicians to improve their patients’ satisfaction, treatment and, all in all, their quality of life.

For these reasons, this study pursued two main objectives: examining the relationship between the psychosocial impact of dental aesthetics and self-esteem and perfectionism, and measuring the influence of gender on the variables under study.

## Material and Methods

A descriptive-analytical cross-sectional study was conducted in a sample of 301 students of the Faculty of Medicine and Dentistry of the University of Valencia, with ages ranging between 18 and 30 years, regardless of gender.

Accepting an α risk of 0.05 and a β risk of under 0.1 in a two-tailed test of the hypothesis, and taking into account a minimum correlation coefficient of 0.2, the necessary sample size was calculated as 288 participants. The field work was carried out during March and April 2016.

A questionnaire composed of three standardised tools (PIDAQ, MPS y RSS) was administered to each of the participants, who were also given an informed consent form and a confidentiality document. The estimated completion time was 15 minutes and the completed questionnaires were collected by the interviewer. The response rate was 79%.

The impact of dental aesthetics was assessed by the PIDAQ (4), which is composed of 23 items divided into 4 dimensions or subscales (1 positive and 3 negative): dental self-confidence (items 1-6), social impact (items 7-14), psychological impact (items 15-20) and aesthetic concern (items 21-23). Each item was rated on a five-point Lickert scale offering the following response options: 1 = totally disagree; 2 = disagree a little; 3 = neither agree nor disagree; 4 = agree a little; and 5 = totally agree. The results for each subscale can be calculated separately by adding up the scores for the responses in that particular dimension or subscale. The overall PIDAQ result is calculated by adding up the values of the four subscales, having previously recoded those for the first subscale (dental self-confidence) from positive to negative to align them with those of the other subscales. The higher the score on adding up the four dimensions, the greater the psychosocial impact of the dental aesthetics of the person interviewed. The PIDAQ has been translated and validated cross-culturally for use in Spain.

The MPS (Multidimensional Perfectionism Scale) developed by Frost *et al.* (18) assesses perfectionism from a perspective that covers several dimensions. It contains 35 items grouped into 6 subscales: personal standards, concern over making mistakes, doubt about one’s actions, parental expectations, parental criticism and organisation (18). The answers scored from 1 to 5 on a Lickert scale with the following options: 1 = completely disagree; 2 = quite disagree; 3 = neither agree nor disagree; 4 = quite agree; and 5 = completely agree.

Self-esteem was measured on the RSS (Rosenberg self-esteem scale) (15), which contains 10 items in a single dimension; 5 are worded positively and 5 negatively. The answers scored from 1 to 5 on a Lickert scale with the following options: 1 = totally disagree; 2 = disagree a little; 3 = neither agree nor disagree; 4 = agree a little; and 5 = totally agree. To calculate the total self-esteem value, the negative items (2, 5, 6, 8 and 9) first have to be recoded positively. After recoding, the values of all the items are added together; the higher the result, the higher the self-esteem.

The data collected through the fieldwork were analysed with SPSS 21.0 software to obtain a descriptive analysis of the items, and the totals and the mean scores of the different scales were calculated. The means and confidence intervals were calculated for the quantitative variables. Student’s t-test was used to assess the differences between means, setting the significance level at *p*<0.05. The associations between the different questionnaires were examined through the Pearson correlation coefficient.

The study was authorized by the University of Valencia’s human research ethics committee (#H1444729526818) and complied with the recommendations of the Helsinki Declaration.

## Results

The sample comprised 301 participants of ages ranging between 18 and 30 years, with a mean age of 20.76 years; 226 were women (75 %) and 75 were men (25 %).

 Table 1 shows a negative correlation (Pearson= -0.387, *p*<0.01) between the total PIDAQ score and self-esteem, indicating a di-vergent or inverse relationship. The correlation of total PIDAQ with perfectionism was found to be positive or convergent (Pearson = 0.281, *p*<0.01). A negative correlation was also found between self-esteem and the dimensions of social impact, psychological impact and aesthetic concern. The relationship between these dimensions and perfectionism was again found to be positive (respectively, Pearson = 0.296, 0.337 and 0.196, *p*<0.01). The relationship between both MPS and RSS and the self-confidence dimension of the PIDAQ should be mentioned, as it differed from that of the other dimensions. As regards the self-esteem questionnaire, a positive relationship with the PIDAQ self-confidence dimension was observed (Pearson = 0.357, *p*<0.01) ( Table 1). On examining the correlations by gender, the same results were obtained ( Table 2).

**Table 1:**
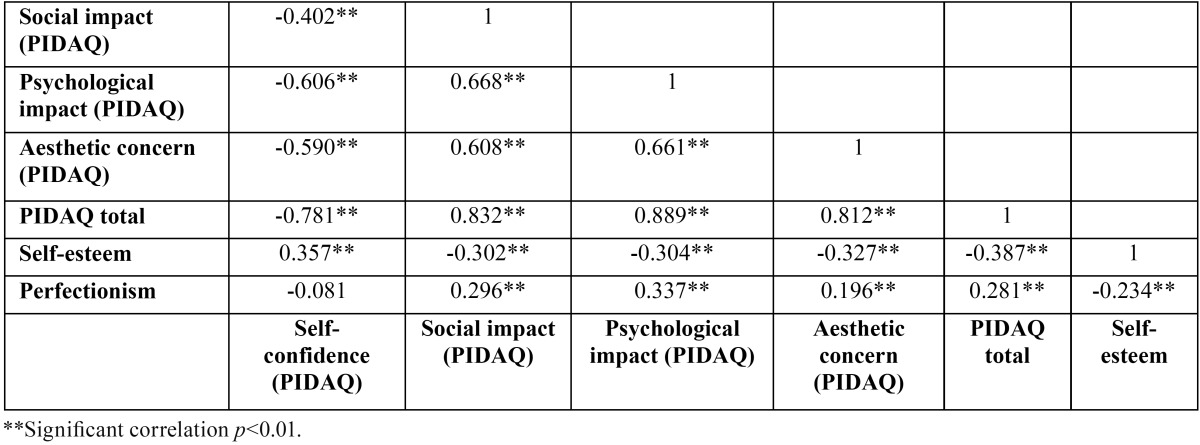
Correlation (Pearson correlation coefficient) between PIDAQ and its subscales and the self-esteem (RSS) and perfectionism (MPS) questionnaires.

**Table 2:**
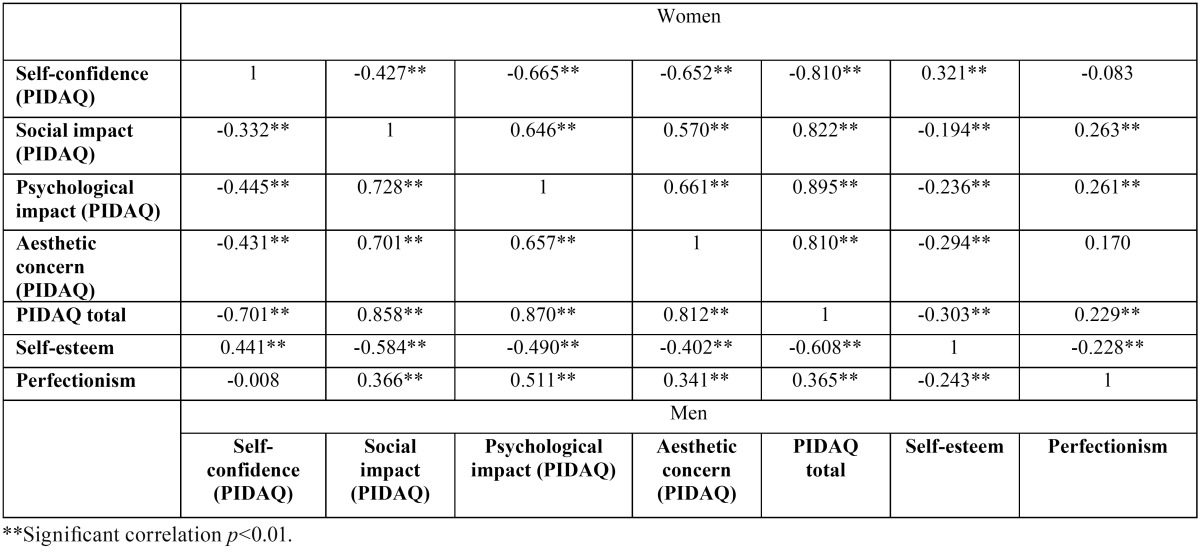
Correlation by gender (Pearson correlation coefficient) between PIDAQ and its subscales and the self-esteem (RSS) and perfectionism (MPS) questionnaires.

**Figure 1:**
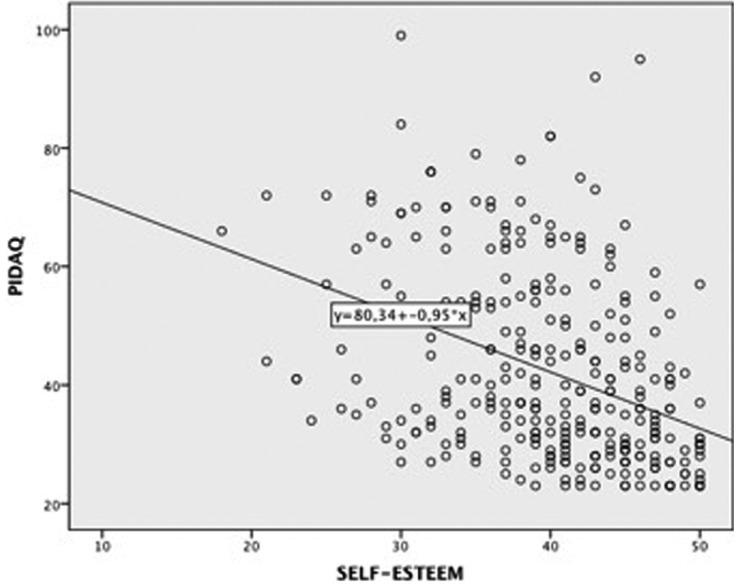
Scatter plot of relationship between PIDAQ and self-esteem.

**Figure 2:**
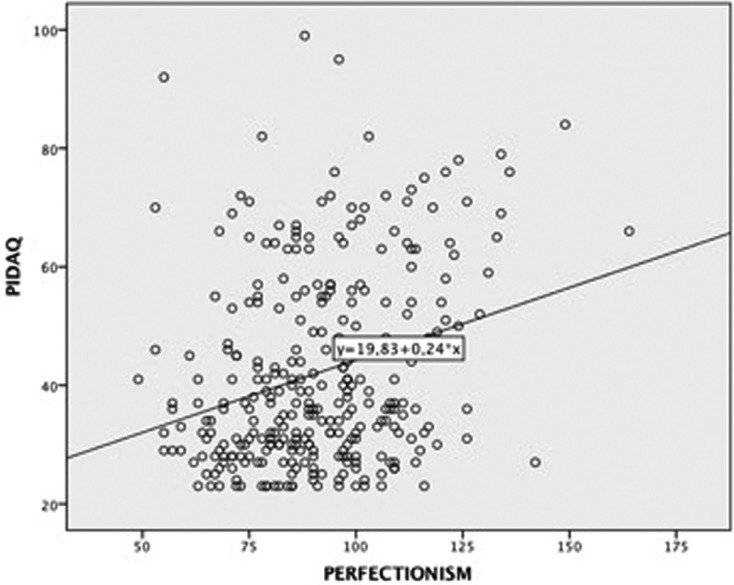
Scatter plot of relationship between PIDAQ and perfectionism.

The associations between PIDAQ and self-esteem and between PIDAQ and perfectionism may be observed in Figures 1 and 2.

Concerning the impact of gender on the study variables, on comparing the scores for the different questionnaires and subscales the only statistically significant differences were found in perfectionism (men 97.4, women 89.1) and confidence (men 22.1, women 23.5) ( Table 3).

**Table 3:**
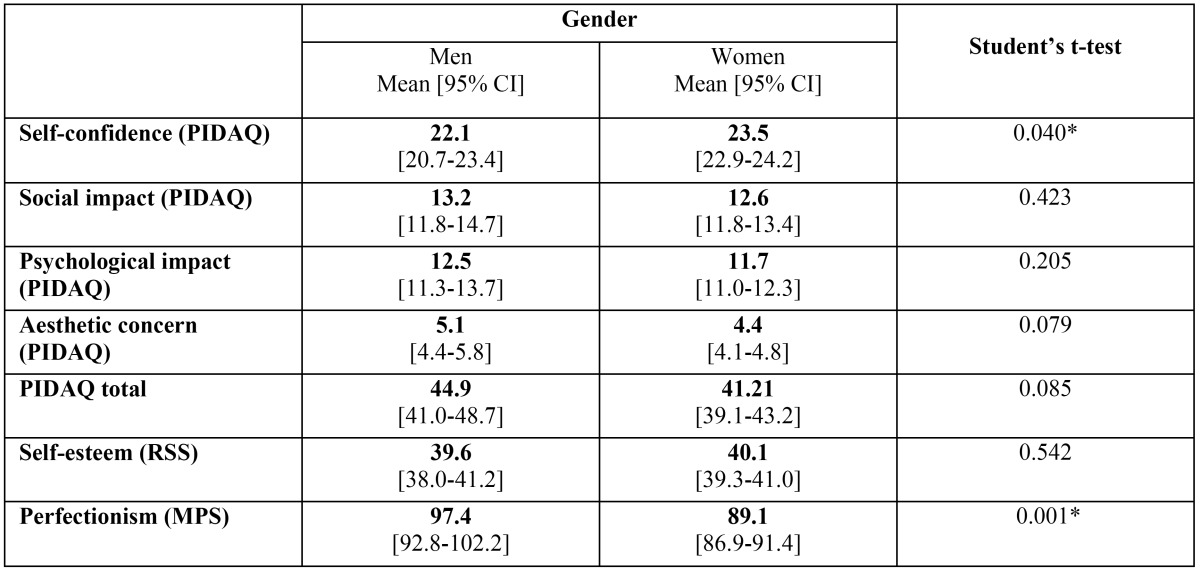
Questionnaire scores by gender.

## Discussion

The present study measured the psychosocial impact of dental aesthetics through the validated Spanish version of the PIDAQ questionnaire, which preserves the original factorial structure, supporting the reliability and validity of these results (4,21,22). Perfectionism was measured with the MPS questionnaire and self-esteem with the Rosenberg questionnaire, both of which have also been validated in Spanish.

The men obtained higher PIDAQ scores, both overall and for the social impact, psychological impact and aesthetic concern dimensions. This means that men were more affected by their dental aesthetics, corroborating a study by Afroz *et al.* (1) in which women were found to be more satisfied with their dental aesthetics. A study by Bellot-Arcís *et al.* (12) differed from the present results, finding a lower psychosocial impact of dental aesthetics among men, although the difference between genders was not significant. A study in adolescents (23) found that this age group places far more importance on its aesthetic appearance. In addition, adolescents present a greater psychosocial impact of dental aesthetics than adults (24,25).

The present study observed a negative correlation between self-esteem and social impact, psychological impact and aesthetic concern, which is corroborated by other studies (16,26). In contrast, the correlation was positive between self-esteem and the PIDAQ questionnaire’s self-confidence subscale; in other words, dental satisfaction has a positive effect on self-esteem (1,16,26).

On examining the results by gender, it was found that the men had lower self-esteem and consequently their dental aesthetics had a greater impact (divergent relationship), whereas in other studies (16) it was the women who placed great importance on dental aesthetics. A study by Jung (27) observed that the girls were generally less self-confident than the boys, in contrast to the present findings. A number of studies have examined the association between aesthetics and gender and have concluded that women are generally more demanding with regard to beauty and aesthetics, more affected by what they consider facial and corporal aesthetic defects and more critical in general regarding everything related to aesthetics (13,28-32). According to several studies (1,12,16), women present a greater psychosocial impact than men. These data are in disagreement with the present study, in which no significant difference in psychosocial impact was observed. However, a study by Afroz *et al.* (1) observed that, as in the present study, a significantly higher number of men than women were concerned about their smile, by how it looked and by how they were seen by others.

Several authors have studied the association between self-esteem and age. Gavric *et al.* (26) found no association but others have observed that self-esteem increases with age in men and decreases in women, from adolescence to adulthood (33).

One bias in the present study could lie in the sample selection, which was limited to a university population. The fact that the majority of the sample were women is due to the greater number of women studying for degrees in medicine and dentistry.

## Conclusions

The students most affected by poor dental aesthetics had lower self-esteem and higher levels of perfectionism. The men exhibited higher levels of perfectionism than the women, while the latter displayed greater self-confidence in their dental aesthetics. Clinicians should pay greater attention to these traits and to their implications for treating these patients.
